# Psychometric data of a questionnaire to measure cyberbullying bystander behavior and its behavioral determinants among adolescents

**DOI:** 10.1016/j.dib.2018.04.087

**Published:** 2018-05-01

**Authors:** A. DeSmet, S. Bastiaensens, K. Van Cleemput, K. Poels, H. Vandebosch, G. Deboutte, L. Herrewijn, S. Malliet, S. Pabian, F. Van Broeckhoven, O. De Troyer, G. Deglorie, S. Van Hoecke, K. Samyn, I. De Bourdeaudhuij

**Affiliations:** a Department of Movement and Sport Sciences, Ghent University, Ghent, Belgium; b Research Foundation Flanders, Brussels, Belgium; c Department of Communication Studies, University of Antwerp, Antwerp, Belgium; d Department of Communication Sciences, Ghent University, Ghent, Belgium; e Department of Computer Science, Vrije Universiteit Brussel, Brussel, Belgium; f Department of Electronics and Information Systems, Ghent University, Ghent, Belgium; g Digital Arts and Media, University College HoWest, Kortrijk, Belgium; h Antwerp Management School, University of Antwerp, Antwerp, Belgium

## Abstract

.This paper describes the items, scale validity and scale reliability of a self-report questionnaire that measures bystander behavior in cyberbullying incidents among adolescents, and its behavioral determinants. Determinants included behavioral intention, behavioral attitudes, moral disengagement attitudes, outcome expectations, self-efficacy, subjective norm and social skills. Questions also assessed (cyber-)bullying involvement. Validity and reliability information is based on a sample of 238 adolescents (M age=13.52 years, SD=0.57). Construct validity was assessed using Confirmatory Factor Analysis (CFA) or Exploratory Factor Analysis (EFA) in Mplus7 software. Reliability (Cronbach Alpha, α) was assessed in SPSS, version 22. Data and questionnaire are included in this article. Further information can be found in DeSmet et al. (2018) [1].

**Specifications table**

**Table:** 

Subject area	*Psychology*
More specific subject area	*Cyberbullying*
Type of data	*Table, text file*
How data was acquired	*Survey*
Data format	*Raw, Analyzed*
Experimental factors	*/*
Experimental features	*/*
Data source location	*Flanders, Belgium*
Data accessibility	*Data and questionnaire are provided within this article*

**Value of the data**

• To our knowledge, this is the first validated questionnaire assessing cyberbullying bystander behavior and its modifiable behavioral determinants based on behavior change theories.
• These data could be useful for researchers to further explore what drives bystander behavior, e.g. in other settings and cultures.
• The questionnaire can be used to evaluate effects on behavior and its determinants of interventions that target bystander behavior and social dynamics of cyberbullying.
• We invite researchers to re-use and further improve on the scale.

## 1 Data

This paper contains psychometric data on a self-report questionnaire for adolescents used to measure their bystander behavior and behavioral determinants in cyberbullying, calculated in a sample of 238 adolescents whose descriptive statistics are provided in Table 1. This is to our knowledge the first validated questionnaire to measure this, and can also be used to assess effects of interventions aiming to change cyberbullying prevalence and its harm by reducing the social reinforcement witnesses give to bullies or victims. Different factor models were tested and fitting indices were computed to find the best fitting solution for each scale. Best fitting solutions per scale and the items they are composed of are shown (Table 2). Data and questionnaire are in supplementary files.

**Table 1:** Participant characteristics.

*Characteristics*	*Baseline sample n*=238
Age	*M*=13.52±0.57
Gender (female)	61.1%
Cyberbullying victimization (% at least 2–3 times/month in past 6 months)	3.5%
Cyberbullying perpetration (% at least 2–3 times/month in past 6 months)	1.7%
Cyberbullying bystanding (% at least 2–3 times/month in past 6 months)	27.4%

**Table 2:** Psychometric properties of behavior and determinant scales.

**Scale**	**Model fit**				
**Behavioral intention (1–5 Likert scale)**	CFI=0.95; Normed *χ*^2^=1.82, *p*<0.01; RMSEA=0.059; SRMR=0.042				
	**Subscale**	**Cronbach α**	**Items** (name in raw data file, questionnaire)	**Rotated factor loading**	**M±SD**
	* Factor 1 ‘negative bystander behavior intention’	*α*=0.60	*Send it to others to laugh at (y4, Q8.3)*	0.76	1.16±0.50
			*Show the bully I thought it was funny (y2, Q8.1)*	0.71	1.32±0.80
			Also send hurtful messages to victim (y5, Q8.4)*	0.38	1.28±0.74
	* Factor 2 ‘positive bystander behavior intention’	*α*=0.74	*Comfort victim (y8, Q8.7)*	0.74	4.21±1.08
			*Give victim advice (y10, Q8.9)*	0.74	3.88±1.04
			*Gather info (y11, Q8.10)*	0.56	3.13±1.17
			*Tell the bully it's not funny (y6, Q8.5)*	0.51	3.64±1.20
			*Ask others not to join in (y7, Q8.6)*	0.49	3.93±1.23
			Show or report to adults for help (y3, Q8.2)*	0.47	3.65±1.20
			Do nothing (negative) (y13, Q8.12)*	-0.45	1.97±1.19
					
**Scale**	**Model fit**				
**Behavioral attitudes (1–7 semantic differential scale)**	CFI=0.92; Normed *χ*^2^=1.99, *p*<0.001; RMSEA=0.065; SRMR=0.058				
	**Subscale**	**Cronbach α Reliability**	**Items** (name in raw data file, questionnaire)	**Standardized estimate (SE)**	**M±SD**
	* Factor 1	*α*=0.85	*Friendly (y15, Q9.2)*	−0.88 (0.03)	6.25±1.55
	**‘Attitudes towards comforting’**		*Bad (negative) (y14, Q9.1)*	0.83 (0.03)	2.22±1.78
			*Brave (y17, Q9.4)*	−0.75 (0.04)	5.83±1.61
	* Factor 2 ‘Attitudes towards giving someone advice’	*α*=0.80	*Friendly (y19, Q10.2)*	−0.87 (0.03)	6.26±1.06
			*Bad (negative) (y18, Q10.1)*	0.74 (0.04)	1.95±1.38
			*Brave (y21, Q10.4)*	−0.73 (0.04)	5.93±1.11
			*Not fun (negative) (y20, Q10.3)*	0.67 (0.04)	2.74±1.48
	* Factor 3 ‘Attitudes towards reporting to adults’	*α*=0.84	*Bad (negative) (y22, Q11.1)*	0.85 (0.03)	2.16±1.61
			*Friendly (y23, Q11.2)*	−0.85 (0.03)	5.70±1.48
			*Not fun (negative) (y24, Q11.3)*	0.69 (0.04)	3.40±1.74
			*Brave (y25, Q11.4)*	−0.65 (0.04)	5.71±1.61
	* Factor 4 ‘Attitudes towards telling the bully it is not cool’	*α*=0.70	*Bad (negative) (y26, Q12.1)*	0.78 (0.06)	2.18±1.77
			*Friendly (y27, Q12.2)*	−0.62 (0.06)	5.60±1.47
			*Not fun (negative) (y28, Q12.3)*	0.61 (0.06)	3.60±1.86
	* Factor 5 ‘Attitudes towards getting back at the bully’	*α*=0.86	*Not fun (negative) (y32, Q13.3)*	0.90 (0.02)	5.82±1.68
			*Bad (negative) (y30, Q13.1)*	0.87 (0.02)	6.00±1.75
			*Friendly (y31, Q13.2)*	−0.74 (0.04)	2.05±1.40
			*Brave (y33, Q13.4)*	−0.62 (0.05)	2.57±1.95
	* Factor 6 ‘Attitudes towards doing nothing’	*α*=0.85	*Bad (negative) (y34, Q14.1)*	0.84 (0.03)	5.73±1.70
			*Not fun (negative) (y36, Q14.3)*	0.84 (0.03)	5.85±1.41
			*Friendly (y35, Q14.2)*	−0.81 (0.03)	2.49±1.60
			*Brave (y37, Q14.4)*	−0.61 (0.05)	2.04±1.53
					
**Scale**	**Model fit**				
**Outcome expectations and self-efficacy (1–5 Likert scale)**	CFI=0.97, Normed *χ*^2^ =1.39, *p*=0.06; RMSEA=0.041; SRMR=0.035				
	**Subscale**	**Cronbach α**	**Items** (name in raw data file, questionnaire)	**Rotated factor loading**	**M±SD**
	* Factor 1 ‘Outcome expectations of assertive defending’	NA	*Standing up for victim ends cyberbullying (y53, Q16.7)*	1.53	2.77±1.03
	* Factor 2 ‘High self-efficacy to comfort or give advice’	*α*=0.72	*Feel well capable of giving victim advice (y57, Q16.11)*	0.85	3.73±0.98
			*Feel well capable of comforting the victim (y56, Q16.10)*	0.74	3.88±1.00
			*By comforting or giving advice, I can make sure the victim is less affected (y51, Q16.5)*	0.51	3.63±1.07
			Standing up for the victim helps the victim (y52, Q16.6)*	0.44	3.55±1.09
			Reporting to adults ends cyberbullying (y54, Q16.8)*	0.39	3.27±1.07
			Know how to end cyberbullying (y55, Q16.9)*	0.36	2.92±1.10
			Not laughing can end cyberbullying (y64, Q16.18)*	0.24	2.84±1.12
	* Factor 3 ‘Low self-efficacy to intervene’	*α*=0.61	*Difficult to comfort victim when I think the victim provoked (y59, Q16.13)*	0.81	2.84±1.18
			*Difficult to comfort the victim when I think it is funny (y58, Q16.12)*	0.49	2.02±1.12
			Difficult to comfort victim when I am not sure of bad intentions of bully (y60, Q16.14)*	0.44	2.84**±**1.15
			Cannot do anything to reduce cyberbullying or its harm (y61, Q16.15)*	0.30	2.49±1.03
					
**Scale**	**Model fit**				
**Subjective norms (1–5 Likert scale)**	CFI=0.95, Normed *χ*^2^=1.76, *p*<0.05; RMSEA=0.057; SRMR=0.043				
	**Subscale**	**Cronbach α**	**Items** (name in raw data file, questionnaire)	**Rotated factor loading**	**M±SD**
	* Factor 1 ‘subjective norm to show positive bystander behavior’	α=0.62	*Friends approve of comforting victim (y39, Q15.2)*	0.77	4.27±1.01
			*Friends would defend victim (y40, 15.3)*	0.67	3.99±1.01
			Friends would approve of joining bully (negative) (y38, Q15.1)*	-0.43	1.35±0.75
			Teachers approve of giving victim advice (y43, Q15.6)*	0.43	4.09±1.05
			Pupils in class disapprove of cyberbullying (y41, Q15.4)*	0.36	4.30±1.10
					
**Scale**	**Model fit**				
**Social skills (1–5 Likert scale)**	CFI=0.95, Normed *χ*^2^=2.21, *p*<0.001; RMSEA=0.072; SRMR=0.048				
	**Subscale**	**Cronbach α Reliability**	**Items** (name in raw data file, questionnaire)	**Standardized estimate (SE)**	**M±SD**
	* Factor 1 ‘Inappropriate social skills’	*α*=0.80	Deliberately hurt others (y74, Q17.6)	0.81 (0.03)	1.31±0.71
			Criticize or nag to bother others (y73, Q17.5)	0.77 (0.04)	1.58±0.91
			Ridicule others (y75, Q17.7)	0.74 (0.04)	1.50±0.82
			Fight/hit when angry (y69, Q17.1)	0.59 (0.05)	2.14±1.16
			Lie to get my way (y72, Q17.4)	0.44 (0.06)	1.94±0.93
	* Factor 2 ‘Appropriate social skills’	*α*=0.79	Feel good when able to help (y77, Q17.9)	0.75 (0.04)	4.39±0.84
			Help a friend in pain (y70, Q17.2)	0.69 (0.05)	4.52±0.69
			Cheer up a friend in pain (y71, Q17.3)	0.69 (0.05)	4.42±0.77
			Ask if I can help (y76, Q17.8)	0.59 (0.05)	3.99±0.85
			Nice to those who are nice to me (y78, Q17.10)	0.58 (0.05)	4.54±0.75
					
**Scale**	**Model fit**				
**Moral disengagement attitudes (1–5 Likert scale)**	No fitting model based on included 3 items, 1 item retained				
	**Subscale**		**Items** (name in raw data file, questionnaire)		**M±SD**
			Youngsters are cyberbullied because they are different (y47, Q16.1)		3.31±1.29
					
**Scale**	**Model fit**				
**Bystander behavior**	No model info available, based on behavioral intention scales				
	**Subscale**		**Items** (name in raw data file, questionnaire)		**%**
	* Subscale 1 ‘Negative bystander behavior’		*Send it to others to laugh at (y94, Q7.3)*		2.9
			*Show the bully I thought it was funny (y92, Q7.1)*		9.6
			*Also send hurtful messages to victim (y95, Q7.4)*		4.4
	* Subscale 2 ‘Positive bystander behavior’		*Comfort victim (y98, Q7.7)*		61.8
			*Give victim advice (y100, Q7.9)*		39.7
			*Gather info (y101, Q7.10)*		26.5
			*Tell the bully it's not funny (y96, Q7.5)*		57.4
			*Ask others not to join in (y97, Q7.6)*		41.2
			*Show or report to adults for help (y93, Q7.2)*		20.0
			*Do nothing (negative) (y103, Q7.12)*		25.0

Standardized estimate for CFA solutions: STDYX=raw coefficient standardized using both latent variable and observed variable variances. Rotated factor loadings: GEOMIN. NA: not applicable. * items with weak corrected item-total correlation *r*<0.40

## 2 Experimental design, materials and methods

Participants in the sample were 8th graders (13–14 year olds) recruited from two schools in Flanders, Belgium. Parents were informed by the school and provided passive consent, youngsters were requested to provide active informed consent. Informed consent was received for 96% of the adolescents, resulting in a sample of 238 youngsters. Data were collected as part of an intervention [1], baseline data (*n*=238) were used for psychometric validation. Ethical approval for the study was provided by the Ethics Committee of the Ghent University Hospital.

Validity of the questionnaire was established in several steps. First, scales were based on existing validated scales, or were constructed following guidelines for the design of theory-based questionnaires on behavior and behavioral determinants. This was the case for: 1) the moral disengagement items that were based on a framework by Hymel et al. [5], and adapted after quantitative research [4]; 2) the social skills scale, that was adapted from the MESSY questionnaire, using five items per scale that were highest loading in previous research [6], [7]; and 3) for questions on behavior and behavioral determinants which were designed using guidelines from behavior change theories on constructing behavior and behavioral determinant scales [2]. These guidelines include e.g. the recommendation to define the target behavior as context- and time specific as possible; to assess positive and negative evaluations of a behavior on bipolar adjective scales (typically 7-point); to base the formulation of items on formative research with users (see for more information: http://people.umass.edu/aizen/pdf/tpb.measurement.pdf). Second, the specific content of the questions was fine-tuned with users via qualitative and quantitative research [3], [4]. For example, adolescents referred to some bystander behavior as considered ‘brave’ or ‘cowardly’. These bipolar adjectives were hence included in the attitude scales. In these two initial steps, the content validity of the questionnaire was established. The current manuscript describes the construct validation and reliability assessment of the questionnaire, examined via Confirmatory or Exploratory Factor Analysis and Cronbach Alpha internal consistency, as recommended in the guidelines for theory-based questionnaire construction on behavior and behavioral determinants [2]. Construct validity refers to the extent to which the scale reflects the theoretical dimensions of the investigated phenomenon, in this case bystander behavior and behavioral determinants.

Bystander behavior questions were only asked to participants who had witnessed a cyberbullying incident in the past month. Theory-based guidelines [2] recommend to assess the behavior as specifically as possible. Formative research with adolescents also showed it was easier for them to discuss behavior referring to a last incident than when referring to a longer time-frame or to a more general concept of behavior. Adolescents were therefore asked if they responded with a certain bystander behavior to the last incident they had witnessed. Formative research showed several types of bystander behavior may occur in combination as response to a single cyberbullying incident [3]. Bystander behavior items were dichotomous (yes/no) and were not factor analyzed, instead they were summed according to the same factorial composition as in behavioral intentions. Definitions of behavioral determinants are provided in DeSmet et al. [1]. Scales were constructed on baseline measures and assessed on their construct validity in Confirmatory Factor Analysis (CFA) or Exploratory Factor Analysis (EFA) using Mplus7 software (Muthén & Muthén). Normed *χ*^2^ (acceptable fit scores ≤3), CFI (Comparative Fit Index, acceptable fit scores ≥0.90), RMSR (Root Mean Square Residual, acceptable fit scores ≤0.08) and SMREA (Root Mean Square Error of Approximation, acceptable fit scores ≤0.08) were used to assess model fit [8]. Reliability (Cronbach Alpha, α) was assessed in SPSS, version 22. Values of 0.60 or above were considered acceptable given the short scales [9]. Factors were trimmed for items which decreased their internal consistency. If after trimming, the factor did not reach satisfactory validity or reliability, one item was retained with either the highest factor loading or with the highest need for improvement. Table 2 presents scales and their psychometric properties. Validity of the scales on behavioral intention scale, attitudes, outcome expectations and self-efficacy, subjective norms, and social skills was good, reaching or exceeding the levels for acceptable fit scores of the Confirmatory or Exploratory Factor Analysis models. No acceptable scale was found for moral disengagement attitudes, where only one item was retained. Reliability of all multi-item scales had a minimal acceptable Cronbach Alpha of 0.60 or higher. Researchers are invited to further improve on certain scales to increase their reliability from an acceptable to a good level. We have marked items (*) with weak item-to-total correlations of *r*<0.40 [10], where future research may wish to modify or replace these items to obtain a more reliable scale.
