# Supplementary material for: Psychometric data of a questionnaire to measure cyberbullying bystander behavior and its behavioral determinants among adolescents
Source: Data Brief. 2018 May 1;18:1588–95. doi: 10.1016/j.dib.2018.04.087 (PMC5998205; doi:10.1016/j.dib.2018.04.087)
Supplement: Supplementary file 2 — Supplementary material [file mmc2.docx]

**Mplus syntax**

1. **Behavioral intentions**

TITLE: RCT;

DATA: FILE is Mplus_scales3.dat;

VARIABLE:

NAMES are y1-y78 ;

USEVARIABLES ARE y2-y8 y10-y11 y13;

MISSING ARE ALL (9999);

ANALYSIS:

ANALYSIS: TYPE = EFA 1 5;

OUTPUT: SAMPSTAT STANDARDIZED MODINDICES(0.0);

1. **Behavioral attitudes**

TITLE: ZDT;

DATA: FILE is Mplus_scales3.dat;

VARIABLE:

NAMES are y1-y78 ;

USEVARIABLES ARE y14-y15 y17-y28 y30-y37;

MISSING ARE ALL (9999);

ANALYSIS:

ESTIMATOR IS ML;

ITERATIONS = 10000;

CONVERGENCE = 0.00005;

MODEL:

f1 BY y14 y15 y17;

f2 BY y18 y19 y20 y21;

f3 BY y22 y23 y24 y25;

f4 BY y26 y27 y28;

f5 BY y30 y31 y32 y33;

f6 BY y34 y35 y36 y37;

OUTPUT: SAMPSTAT STANDARDIZED MODINDICES(0.0);

1. **Outcome expectations and self-efficacy**

TITLE: RCT;

DATA: FILE is Mplus_scales3.dat;

VARIABLE:

NAMES are y1-y78 ;

USEVARIABLES ARE y51-y61 y64;

MISSING ARE ALL (9999);

ANALYSIS:

ANALYSIS: TYPE = EFA 1 5;

OUTPUT: SAMPSTAT STANDARDIZED MODINDICES(0.0);

1. **Subjective norm**

TITLE: RCT;

DATA: FILE is Mplus_scales3.dat;

VARIABLE:

NAMES are y1-y78 ;

USEVARIABLES ARE y38-y43 y45;

MISSING ARE ALL (9999);

ANALYSIS:

ANALYSIS: TYPE = EFA 1 4;

OUTPUT: SAMPSTAT STANDARDIZED MODINDICES(0.0);

1. **Social skills**

TITLE: RCT;

DATA: FILE is Mplus_scales3.dat;

VARIABLE:

NAMES are y1-y78 ;

USEVARIABLES ARE y69-y78;

MISSING ARE ALL (9999);

ANALYSIS:

ESTIMATOR IS ML;

ITERATIONS = 10000;

CONVERGENCE = 0.00005;

MODEL:

f1 BY y69 y72 y73 y74 y75;

f2 BY y70 y71 y76 y77 y78

OUTPUT: SAMPSTAT STANDARDIZED MODINDICES(0.0);
