# Supplementary material for: Psychometric data of a questionnaire to measure cyberbullying bystander behavior and its behavioral determinants among adolescents
Source: Data Brief. 2018 May 1;18:1588–95. doi: 10.1016/j.dib.2018.04.087 (PMC5998205; doi:10.1016/j.dib.2018.04.087)
Supplement: Supplementary file 3 — Supplementary material [file mmc3.docx]

**The following questions handle bullying. We call something bullying if**

- **People do or say something mean or hurtful more than once**
- **The bully has the intention to make others feel bad**
- **The person who is bullied has a difficult time defending him- or herself**

**We do not call it bullying when friends tease each other or fight.**

**Bullying can also take place using electronic media, such as Internet or mobile phones: cyberbullying. Someone who cyberbullies can for example: send nasty messages via SMS or chat, distribute hurtful pictures via Internet or mobile phone, post insulting reactions on a social networking site (such as Facebook), and spread gossip via a website. The following questions ONLY handle cyberbullying, this means bullying via Internet or mobile phone.**

1. **How often were you bullied via the Internet or mobile phone in the past 6 months (=cyberbullying?)**

- I was not cyberbullied in the past 6 months via Internet or mobile phone
- Once or twice in the past 6 months
- 2 or 3 times in the past month
- About once a week
- Several times per week

1. **How often were you bullied via the Internet or mobile phone in the past month (=cyberbullying?)**

- I was not cyberbullied in the past month via Internet or mobile phone
- Once in the past month
- 2 or 3 times in the past month
- About once a week
- Several times per week

1. **How often did you bully others via the Internet or mobile phone in the past 6 months (=cyberbullying?)**

- I did not cyberbully anyone in the past 6 months via Internet or mobile phone
- Once or twice in the past 6 months
- 2 or 3 times in the past month
- About once a week
- Several times per week

1. **How often did you bully others via the Internet or mobile phone in the past month (=cyberbullying?)**

- I did not cyberbully anyone in the past month via Internet or mobile phone
- Once in the past month
- 2 or 3 times in the past month
- About once a week
- Several times per week

1. **How often did you see that others were bullied via the Internet or mobile phone in the past 6 months (=cyberbullying?)**

- Never in the past 6 months
- Once or twice in the past 6 months
- 2 or 3 times in the past month
- About once a week
- Several times per week

1. **How often did you see that others were bullied via the Internet or mobile phone in the past month (=cyberbullying?)**

- Never in the past 6 months
- Once in the past month
- 2 or 3 times in the past month
- About once a week
- Several times per week

If you answered ‘never’ to question 6, please skip to question 8

1. **What did you do the last time in the past month that you noticed someone else was being bullied via Internet or mobile phone?**

|  | **Yes** | **No** |
| --- | --- | --- |
| Q7.1. I made it clear to the bully that I thought it was funny (e.g. by clicking on ‘like’ or saying something about it) |  |  |
| Q7.2. I showed it to or told an adult to look for help |  |  |
| Q7.3. I forwarded it to others to laugh about it |  |  |
| Q7.4. I responded by also sending hurtful messages about or to the victim |  |  |
| Q7.5. I told the bully I thought it was not funny or cool |  |  |
| Q7.6. I told or asked friends to not join in |  |  |
| Q7.7. I comforted the one who was getting bullied (e.g. told them not to let it get to them, that I don’t agree with the bully) |  |  |
| Q7.8. I got back at the bully or swore at him/her* |  |  |
| Q7.9. I gave the person who was bullied advice on how to deal with the bullying, to help him/her |  |  |
| Q7.10 I collected more information on what happened among the bully, the victim or their friends |  |  |
| Q7.11. I told the person who has getting bullied to adjust and try to fit in to end the bullying* |  |  |
| Q7.12. None of the above, I did nothing (I just ignored it) |  |  |

* not used in scale composition

1. **What do you expect you will do next time you notice someone else is being bullied via Internet or mobile phone?**

|  | Fully disagree | Rather disagree | Do not agree, do not disagree | Rather agree | Fully agree |
| --- | --- | --- | --- | --- | --- |
| Q8.1. I will make it clear to the bully that I think it was funny (e.g. by clicking on ‘like’ or saying something about it) |  |  |  |  |  |
| Q8.2. I will show it to or tell an adult to look for help |  |  |  |  |  |
| Q8.3. I will forward it to others to laugh about it |  |  |  |  |  |
| Q8.4. I will respond by also sending hurtful messages about or to the victim |  |  |  |  |  |
| Q8.5. I will tell the bully I think it is not funny or cool |  |  |  |  |  |
| Q8.6. I will tell or ask friends to not join in |  |  |  |  |  |
| Q8.7. I will comfort the one who is getting bullied (e.g. tell them not to let it get to them, that I don’t agree with the bully) |  |  |  |  |  |
| Q8.8. I will get back at the bully or swear at him/her* |  |  |  |  |  |
| Q8.9 I will give the person who is bullied advice on how to deal with the bullying, to help him/her |  |  |  |  |  |
| Q8.10. I will collect more information on what happened among the bully, the victim or their friends |  |  |  |  |  |
| Q8.11. I will tell the person who is getting bullied to adjust and try to fit in to end the bullying* |  |  |  |  |  |
| Q8.12. None of the above, I will do nothing (I will just ignore it) |  |  |  |  |  |

* not used in scale composition

**For the following questions, think of the last case of cyberbullying that you have witnessed. Indicate in the grids how you feel about the following reactions to cyberbullying. The closer the dot is placed to a word, the more you think it applies.**

**E.g. how do you feel about chocolate? If you think chocolate is a bit tasty, you color the fifth dot.**

|  | 1 | 2 | 3 | 4 | 5 | 6 | 7 |  |
| --- | --- | --- | --- | --- | --- | --- | --- | --- |
| Disgusting |  |  |  |  |  |  |  | Tasty |

1. **To comfort someone who is being bullied via Internet of mobile phone, is according to me…**

|  | 1 | 2 | 3 | 4 | 5 | 6 | 7 |  |
| --- | --- | --- | --- | --- | --- | --- | --- | --- |
| Q9.1. Good |  |  |  |  |  |  |  | Bad |
| Q9.2. Mean |  |  |  |  |  |  |  | Friendly |
| Q9.3. Fun |  |  |  |  |  |  |  | Not fun |
| Q9.4. Cowardly |  |  |  |  |  |  |  | Brave |

1. **To give someone advice who is being bullied via Internet of mobile phone, is according to me…**

|  | 1 | 2 | 3 | 4 | 5 | 6 | 7 |  |
| --- | --- | --- | --- | --- | --- | --- | --- | --- |
| Q10.1. Good |  |  |  |  |  |  |  | Bad |
| Q10.2. Mean |  |  |  |  |  |  |  | Friendly |
| Q10.3. Fun |  |  |  |  |  |  |  | Not fun |
| Q10.4. Cowardly |  |  |  |  |  |  |  | Brave |

1. **To report it to adults when someone is being bullied via Internet of mobile phone, is according to me…**

|  | 1 | 2 | 3 | 4 | 5 | 6 | 7 |  |
| --- | --- | --- | --- | --- | --- | --- | --- | --- |
| Q11.1. Good |  |  |  |  |  |  |  | Bad |
| Q11.2. Mean |  |  |  |  |  |  |  | Friendly |
| Q11.3. Fun |  |  |  |  |  |  |  | Not fun |
| Q11.4. Cowardly |  |  |  |  |  |  |  | Brave |

1. **To tell the bully that you don’t like it or ask him/her to stop when someone is being bullied via Internet of mobile phone, is according to me…**

|  | 1 | 2 | 3 | 4 | 5 | 6 | 7 |  |
| --- | --- | --- | --- | --- | --- | --- | --- | --- |
| Q12.1. Good |  |  |  |  |  |  |  | Bad |
| Q12.2. Mean |  |  |  |  |  |  |  | Friendly |
| Q12.3. Fun |  |  |  |  |  |  |  | Not fun |
| Q12.4. Cowardly |  |  |  |  |  |  |  | Brave |

1. **To get back at the bully or bully him/her back when someone is being bullied via Internet of mobile phone, is according to me…**

|  | 1 | 2 | 3 | 4 | 5 | 6 | 7 |  |
| --- | --- | --- | --- | --- | --- | --- | --- | --- |
| Q13.1. Good |  |  |  |  |  |  |  | Bad |
| Q13.2. Mean |  |  |  |  |  |  |  | Friendly |
| Q13.3. Fun |  |  |  |  |  |  |  | Not fun |
| Q13.4. Cowardly |  |  |  |  |  |  |  | Brave |

1. **To do nothing when someone is being bullied via Internet of mobile phone, is according to me…**

|  | 1 | 2 | 3 | 4 | 5 | 6 | 7 |  |
| --- | --- | --- | --- | --- | --- | --- | --- | --- |
| Q14.1. Good |  |  |  |  |  |  |  | Bad |
| Q14.2. Mean |  |  |  |  |  |  |  | Friendly |
| Q14.3. Fun |  |  |  |  |  |  |  | Not fun |
| Q14.4. Cowardly |  |  |  |  |  |  |  | Brave |

1. **Please indicate to which extent you agree with the following statements about yourself and others**

|  | Fully disagree | Rather disagree | Do not agree, do not disagree | Rather agree | Fully agree |
| --- | --- | --- | --- | --- | --- |
| Q15.1. My friends would think it’s a good thing if I would join in bullying someone on the Internet or via mobile phone |  |  |  |  |  |
| Q15.2. My friends would think it’s a good thing if I comfort someone who is being bullied on the Internet or via mobile phone |  |  |  |  |  |
| Q15.3. My friends would defend the victim when they see someone getting bullied on the Internet or via mobile phone |  |  |  |  |  |
| Q15.4. Pupils in my class disapprove of cyberbullying, in my class we don’t think this is ok |  |  |  |  |  |
| Q15.5. Most people who are important to me would think it’s a bad thing if I would ignore the bullying that happens via Internet or mobile phone |  |  |  |  |  |
| Q15.6. My teachers would approve if I give someone who is getting cyberbullied advice |  |  |  |  |  |
| Q15.7. My parents would think it’s a bad thing if I tell the bully to stop |  |  |  |  |  |
| Q15.8. My teachers don’t care if I would join in with the cyberbullying, it leaves them indifferent |  |  |  |  |  |
| Q15.9. Sometimes I feel obliged to join in the bullying on the Internet or via mobile phone, even if I don’t really want to myself |  |  |  |  |  |

1. **To what extent do you agree with the following statements?**

|  | Fully disagree | Rather disagree | Do not agree, do not disagree | Rather agree | Fully agree |
| --- | --- | --- | --- | --- | --- |
| Q16.1. Youngsters are being cyberbullied because they are different |  |  |  |  |  |
| Q16.2. Some youngsters are being cyberbullied because they hurt others |  |  |  |  |  |
| Q16.3. If some youngsters wouldn’t cry or give in so quickly, they would be cyberbullied less |  |  |  |  |  |
| Q16.4. By doing nothing, I at least don’t make it any worse for the victim |  |  |  |  |  |
| Q16.5. Comforting the victim or giving advice, can help the victim to feel the cyberbullying is not so bad |  |  |  |  |  |
| Q16.6. When I take sides with the victim by saying something to the bully, this helps the victim |  |  |  |  |  |
| Q16.7. When I take sides with the victim by saying something to the bully, this ends the cyberbullying |  |  |  |  |  |
| Q16.8. By reporting it to adults when I see cyberbullying taking place, this ends the cyberbullying |  |  |  |  |  |
| Q16.9. I know how I can make cyberbullying stop when I see it happening |  |  |  |  |  |
| Q16.10. I feel well capable of comforting the victim |  |  |  |  |  |
| Q16.11. I feel well capable of giving the victim advice on how to handle cyberbullying |  |  |  |  |  |
| Q16.12. I find it difficult to comfort the one who is being cyberbullied if I actually think it is funny |  |  |  |  |  |
| Q16.13. Sometimes I think the victim provoked it. In this case I find it difficult to comfort or support the victim |  |  |  |  |  |
| Q16.14. I find it difficult to comfort the one who is being cyberbullied if I am not sure that the bullying was done with bad intentions |  |  |  |  |  |
| Q16.15. There is nothing I can do anyway to reduce the cyberbullying or make it less harmful |  |  |  |  |  |
| Q16.16. Cyberbullying can lead to serious problems, such as depression, sleeping disorders, or even suicide |  |  |  |  |  |
| Q16.17. To laugh at it, to ‘like’ it or forward it, is just as bad as the cyberbullying itself |  |  |  |  |  |
| Q16.18. By not laughing at it, I can make the cyberbullying stop |  |  |  |  |  |
| Q16.19. When someone laughs at it, the victim feels even worse |  |  |  |  |  |
| Q16.20. By not joining in with the bully, you increase your popularity |  |  |  |  |  |
| Q16.21. Cyberbullying is never right or justified, even if the victim is weird or someone I don’t like |  |  |  |  |  |
| Q16.22. By telling the victim how to behave differently to make the cyberbullying stop, the victim feels even worse |  |  |  |  |  |

1. **To what extent does this apply to you?**

|  | **1.**  **Not at all** | **2** | **3** | **4** | **5**  **Very much** |
| --- | --- | --- | --- | --- | --- |
| Q17.1. I hit or fight when I am angry |  |  |  |  |  |
| Q17.2. I help a friend who is in pain |  |  |  |  |  |
| Q17.3. I cheer up a friend who is in pain |  |  |  |  |  |
| Q17.4. I lie to get what I want |  |  |  |  |  |
| Q17.5. I nag or criticize other people to get them angry |  |  |  |  |  |
| Q17.6. I hurt other people’s feelings on purpose (I make them sad on purpose) |  |  |  |  |  |
| Q17.7. I ridicule others |  |  |  |  |  |
| Q17.8. I ask if I can help others |  |  |  |  |  |
| Q17.9. I feel good when I have been able to help others |  |  |  |  |  |
| Q17.10. I am nice to those who are nice to me |  |  |  |  |  |
